# Spontaneous separation of large-spin Fermi gas in the harmonic trap: a density functional study

**DOI:** 10.1038/srep31776

**Published:** 2016-08-23

**Authors:** Zongli Sun, Qiang Gu

**Affiliations:** 1 Department of Physics, University of Science and Technology Beijing, Beijing 100083, China; 2 Science and Technology College, North China Electric Power University, Baoding, 071051, China

## Abstract

The component separation of the trapped large-spin Fermi gas is studied within density functional theory. The ground state and ferromagnetic transition in the gas, with and without the spin mixing collision, are calculated. In the absence of spin mixing, two patterns of separation are observed as the interaction between atoms increases, whereas only one of them corresponds to a ferromagnetic transition. The phase diagram suggests that the pattern which the system chooses depends on the interaction strength in the collision channels. With the presence of spin mixing, the distribution of phase region changes because of the interplay between different collision channels. Specifically, the spin exchange benefits the FM transition, while it suppresses the component separation of CS-II pattern.

Itinerant ferromagnetism in metals originates from the quantum correlation between de-localized electrons^1^. Stoner interpreted it as a result of the competition between the short-range repulsion and kinetic energy^2^. Theoretically, this mechanism provides satisfactory explanation for the emergence of the ferromagnetism (FM) transition^3^. However, verification of the Stoner theory in experiment is rather difficult, due not only to the complexity of the interaction in metals, but to the difficulty in manipulating these interactions. Fortunately, cold atoms provide an ideal test-bed to perform a direct examination of the Stoner model^4^,^5^,^6^,^7^,^8^,^9^,^10^,^11^. Compared to the electrons in metals, the ultra-cold Fermi atom gas provides a cleaner model system for the experimental verification of the Stoner model. Moreover, the interaction between atoms can be tuned flexibly^12^,^13^, owing to the success of the Feshbach resonance technique^14^,^15^.

Repulsive Fermi gas with spin-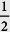 represents a preferred analogue of the electron gas and is relatively convenient in experiment preparation^16^,^17^,^18^,^19^. By monitoring the energy and volume, the MIT group achieved some signatures of the FM transition in ^6^Li atom gas^20^. Although it still remains controversial whether the observation is consistent with the experimental evidence for an FM transition^21^, the experiment has greatly stimulated research interest in the itinerant ferromagnetism in cold atoms. More recently, it was also found that the itinerant FM state is usually prevented by a rapid decay into bound pairs due to the three-body collisions^22^.

Cold atom gas is more than a test-bed for the original Stoner model. It enriches the physics regarding the Stoner model and the itinerant ferromagnetism. Some research has been devoted to the mass-imbalanced two-component Fermi gas. It is pointed out that the phase separation in such systems can be driven by a large mass difference, but not necessarily by the strong repulsions^23^. In addition, the broken SU(2) symmetry in the mixture can deliver unique experimental signatures for the FM phase^24^,^25^. The large-spin Fermi gas (LSFG) is another unique system distinct from the electron gas. It contains more components and thus more interaction channels between atoms^26^,^27^,^28^,^29^,^30^,^31^,^32^. The interacting strength of different collision channels could be different, which may break, at least partially, the symmetry of the Hamilton, and thus facilitates the formation of the FM phase. Note that unlike the case of the mass-imbalanced mixture, the symmetry breaking of the Hamilton takes place only in the interaction terms for the LSFG.

In this paper we concentrate on the FM transition in the LSFG. Recently, considerable effort has been devoted to the spin dynamics^33^,^34^,^35^,^36^ and Mott-insulator transformation^37^,^38^ in the LSFG. To our best knowledge, however, the study related to FM transition in LSFG is rare. We expect that the complex interactions could result in a variety of phenomena related to the FM transition. Especially, the spin mixing collision channel permits the incoming and outgoing spin states to be different^39^, which does not appear in the spin-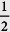 system. Therefore, the LSFG may display novel phase behaviors or new patterns for the formation of domain and texture, which can cast new lights on the understanding about the FM transition in quantum gas.

## Model and Theory

In this work, we consider a confined LSFG, which consists of atoms with hyperfine spin 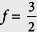. The spinor character implies that the Fermi gas can be treated as a four-components mixture with pseudo-spin 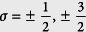. Accordingly, the Hamiltonian of the system can be given by:


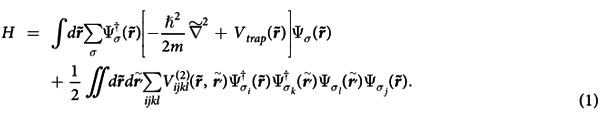


Here 
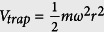
 is the spin-independent external potential applied by the trap and *V*^(2)^ the spin-dependent pair potential between atoms. Ψ_*σ*_ is the atomic field annihilation operator associating the hyperfine spin state 
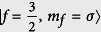
. Note that in Eq. (1), the tilde is used to distinguish the qualities from their reduced forms, which will be defined below. The factor 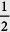 is added in the second term to avoid overcounting in the summation. In the low energy regime, the *s*-wave scattering dominates the collision processes and the interaction can be modeled by the contact potential, i.e., 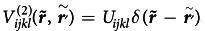. The coupling coefficient *U*_*ijkl*_ can be obtained from the two-body interaction model 
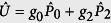
, with the projection operator 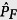. Moreover, 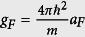 with *a*_*F*_ denoting the *s*-wave scattering length in total *F* spin channel. Note that only the even values of *F* are relevant due to the symmetry of the wave functions in the *s*-wave channel.

**Figure 1:**
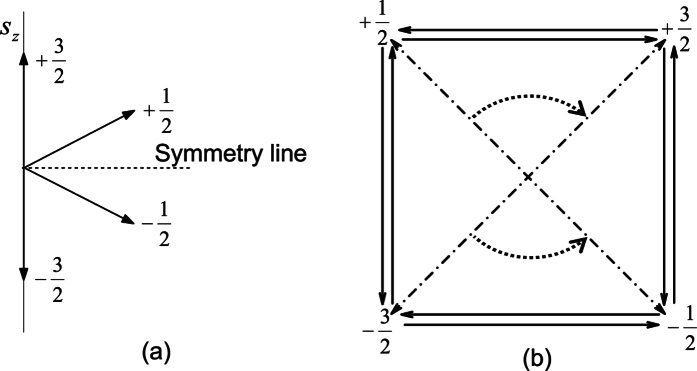
(a) Sketch of the spin orientation for each spin species in the spin-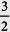 Fermi system. The dash line stands for a symmetry line perpendicular to the *z* axial. (**b**) Schematic of the collision channels in the spinor Fermi gas with 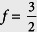. The solid lines and the dash-dotted diagonal lines correspond respectively to collision channels with the coupling strength 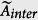, 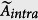, while the dotted arcs to those with 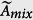.

In order to specify the contribution from different collision channels, it is convenient to decompose the interaction Hamiltonian into three parts, i.e., *H*_*inter*_, *H*_*intra*_ and *H*_*mix*_:


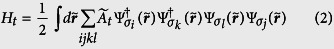


with *t* = *inter, intra, mix* and *A*_*t*_ the corresponding coupling parameters. The possible collision channels in the spin-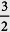 Fermi gas have been shown in Fig. 1. Specifically, *H*_*inter*_ and *H*_*intra*_ describe respectively the contribution from the atom collision between atoms of the symmetrical and asymmetrical spin orientation, while *H*_*mix*_ takes into account the contribution form the spin mixing collision. Projecting the two-body interaction to the total spin space, one obtains the coupling coefficients in terms of *g*_*F*_^40^: 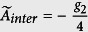, 
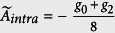
, 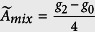. Note that in this work, we choose 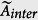 and 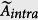 as the independent parameters, which give the third one by 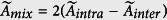. Interaction between atoms of the same spin orientation is absent because of the Pauli exclusion.

On the theoretical side, density functional theory^41^ is a powerful tool which of several theoretical superiorities, including exact mathematical framework and inexpensive numerical cost. With the proper approximation for the exchange-correlation energy, good performance has been shown in its application to the spin-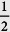 Fermi gas^42^,^43^,^44^. Among the available treatments, local density approximation (LDA) is favored due to its relative simplicity and efficiency in prediction of FM transition. Especially for the two-components trapped Fermi gas, the critical scattering length predicted by LDA is in good agreement with that obtained from experiments^8^. In fact, the ignored surface tension term in LDA may be important, especially near the boundary of the atom cloud. However, it depends largely on the atom number in the cloud. For a gas with large atom number, the inclusion of surface effect leads to nonsignificant difference in its comparison with LDA results^44^. Therefore, it is believed that the application of LDA in LSFG can also provide qualitative predictions for the FM transition, though the correlation in it should be even more complex.

Prior to performing calculations for the ground state, we construct firstly the density functional for the LSFG in the following form:


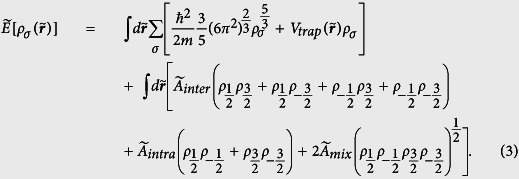


Obviously, the kinetic contribution is given in the Thomas-Fermi form, which treats the kinetic energy only as a correction. Actually, the validity of this approximation is restricted by the criterion 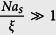, with *N, a*_*s*_, 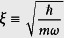 are respectively the number of atoms, the *s*-wave scattering length and the quantum mechanical length scale for the oscillator. Thence, the Thomas-Fermi approximation for the kinetic contribution can be effective so long as *N* is large enough. The detail for the construction of the interaction energy functional is described in the section Method.

For simplicity in the further calculation, it is necessary to transform the related qualities in Eq. (3) to their reduced forms. Here we introduce the parameters *c*_1_ ~ *c*_6_, which satisfy: 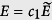, 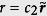, *n* = *c*_3_*ρ*, 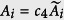, 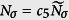, 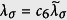 with 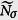 and 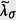 are respectively the particle number and chemical potential of spin-*σ* component. These six above-defined parameters can reduce the total energy to the following form:


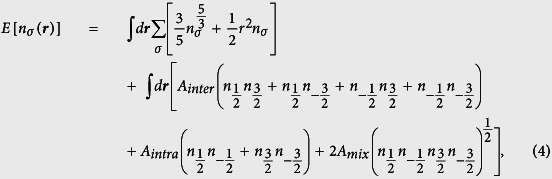


with 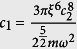, 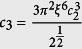, 
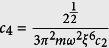
, 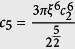, 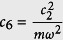. To ensure the conservation of the particle number of each spin component, the Lagrange multiplier *λ*_*σ*_ should be introduced, which relates to the chemical potential of the spin-*σ* component. Therefore, the density of each component in their ground state can be determined by the Euler equations derived from the variational principle:


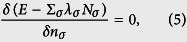


with 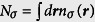 the reduced particle number of the spin *σ*-component.

## Results and Discussion

From the energetic point of view, component separation and spin mixing are two ways for the LSFG to lower its total energy. As in spin-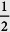 Fermi gas, the competition between repulsive interaction and kinetic energy is responsible for the FM transition. The former tends to induce polarization, while the latter prefers to equally populate each component in local regions. In the LSFG, more collision channels between different spin components are opened, which may supply new alternatives to lower the total energy. In the following, the FM transition and spin mixing process are studied through the calculation of the ground density profile of each component. Note that in this work, the particle number of each component is set as 10^6^, which can be reduced to *N*_*σ*_ = 0.1 in the calculation.

### Coupling with *A*_*inter*_ = *A*_*intra*_ and *A*_*mix*_ = 0

For simplicity, calculations are firstly performed for the LSFG with regular collision channels. Specifically, in the absence of spin mixing collision, the interaction energy *E*_*int*_ can be given by:


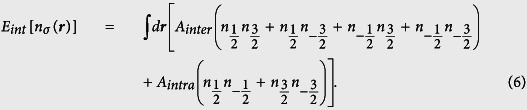


Note that with further simplification of *A*_*inter*_ = *A*_*intra*_, the energy functional in Eq. (6) shows SU(4) symmetry, which indicates that there should exist degenerate ground states because the total energy keeps unchanged as the order of the subscripts 
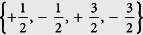
 is arbitrarily exchanged.

**Figure 2:**
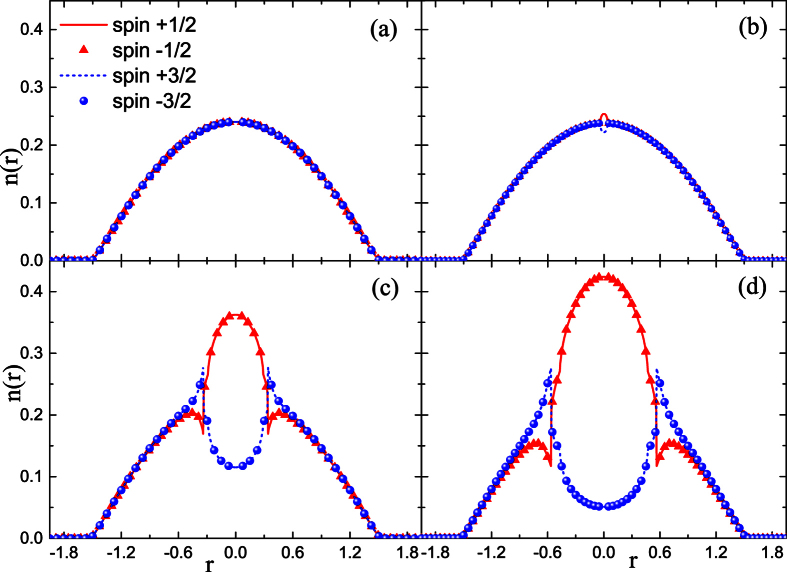
Density profiles of spin components in the LSFG under different conditions of strength parameters. From (**a–d**), *A*_*inter*_ = *A*_*intra*_ = 1.05, 1.08, 1.10, 1.15, respectively.

The results in Fig. 2 suggest that the coupling strength is the critical factor for the occurrence of CS, which does not appear until the coupling is as strong as *A*_*inter*_ = *A*_*intra*_ = 1.08. This is similar to the spin-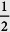 Fermi gas, whose FM transition occurs at the Stoner point. As expected, the CS state is enhanced as the coupling strength is increased. Note that the density profile shown in Fig. 2 is only one of the degenerate states of the LSFG because our further calculation verifies that all of the possible degenerate states have the same ground energy. Therefore, it is concluded that in the case of *A*_*inter*_ = *A*_*intra*_, the LSFG behaves like a two-components system, because the energy consumption in the FM transition is the same as that in the CS between spins with asymmetric orientation.

### Coupling with *A*_*inter*_ ≠ *A*_*intra*_ and *A*_*mix*_ = 0

**Figure 3:**
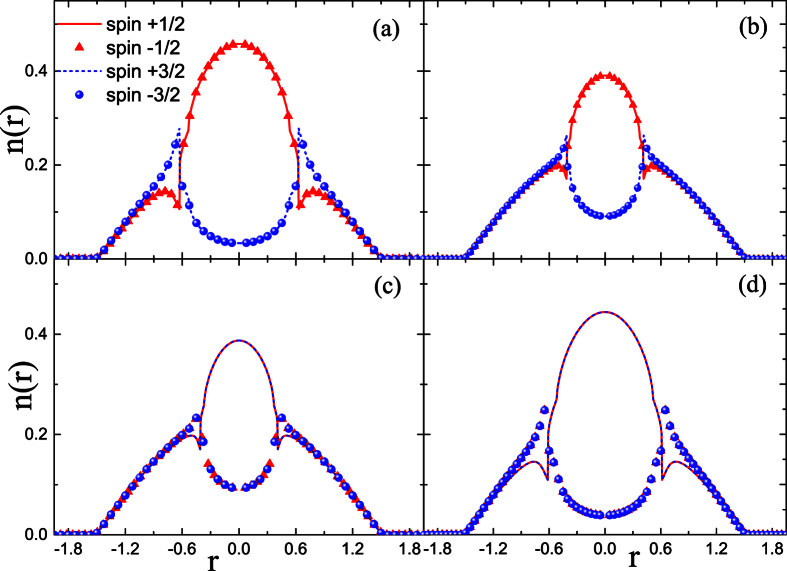
Density profiles of spin components in the LSFG under the condition of *A*_*inter*_ = 1.10. In (**a–d**), the coupling parameter is set as *A*_*intra*_ = 1.04, 1.09, 1.11, 1.16, respectively.

**Figure 4:**
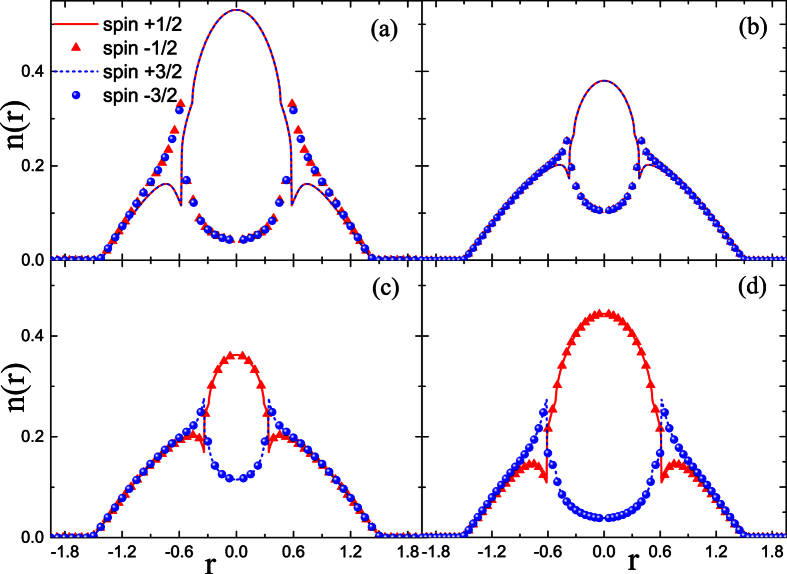
Density profiles of spin components in the LSFG under the condition of *A*_*intra*_ = 1.10. In (**a–d**), the coupling parameter is set as *A*_*inter*_ = 0.6, 1.05, 1.10, 1.13, respectively.

To get more information about the influence of coupling strength on the component separation, further calculations are performed in the case of *A*_*inter*_ ≠ *A*_*intra*_. It is also hoped that the results in this section can provide information which helps us to understand the effect of spin mixing on the FM transition. Obviously, the hidden SU(4) symmetry has been broken due to the difference between *A*_*inter*_ and *A*_*intra*_. The results in Figs 3 and 4 show respectively the influence of *A*_*intra*_(*A*_*inter*_) on the CS for the given coupling parameter *A*_*inter*_(*A*_*intra*_) = 1.10.

As specifically shown in Fig. 3, when *A*_*intra*_ is relatively small, the CS occurs between atoms with asymmetrical spin orientation, i.e., 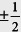 and 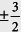 species. This pattern of CS is denoted by CS-I, in which the two components with symmetrical spin orientation always have the same local population. Moreover, with the enhancement of *A*_*intra*_, this type of CS is suppressed, as shown by Fig. 3(b). However, when *A*_*intra*_ is large enough, the separation occurs between atoms with symmetrical spin orientation, i.e., 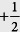
(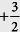) and 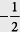
(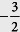), which is denoted by CS-II. Unlike the case of CS-I, the further increase of *A*_*intra*_ enhances the CS-II pattern, as compared by Fig. 3(c,d). The difference between these two patterns of CS can be understood from Eq. (6), which indicates that the coupling strength *A*_*inter*_ relates to the collision between spins with asymmetrical orientation, while *A*_*intra*_ to that between spins with symmetrical orientation.

Note that though two patterns of CS state have been observed during the variation of the coupling parameters, only the CS-II pattern corresponds to the FM state because the non-zero local spin magnetic moment is formed only in this case. As a comparison, the ground states for the given *A*_*intra*_ and different *A*_*inter*_ are also calculated in Fig. 4. The separation with patterns of CS-I and CS-II are also observed during the adjustment of *A*_*inter*_.

### Phase diagram of the LSFG with *A*_*mix*_ = 0

**Figure 5:**
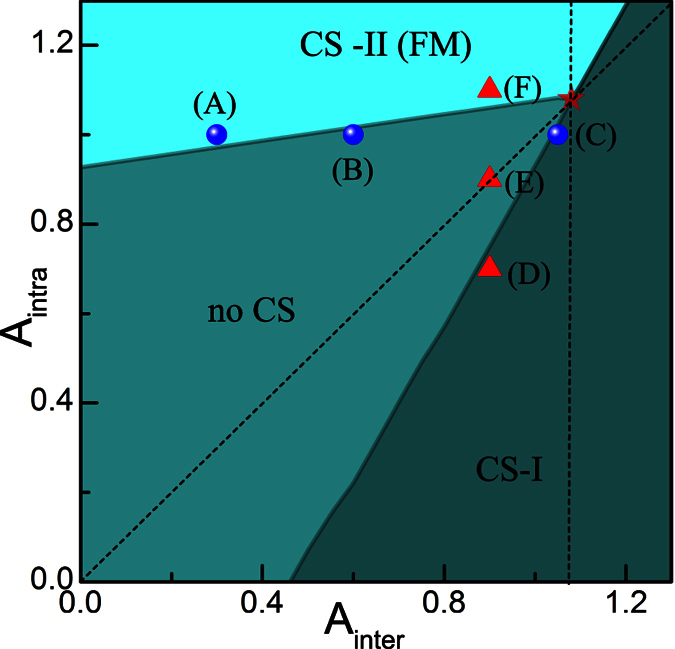
Phase diagram the LSFG without spin mixing. The star represents the triple point where different phases join. The diagonal dash line is the dividing line corresponding to *A*_*inter*_ = *A*_*intra*_, while the vertical dash line passes through the triple-point.

To obtain more comprehensive understanding about the occurrence of CS, we have calculated the phase diagram in Fig. 5, which depicts the critical coupling strength that induces the CS state in the LSFG. In the calculation, we choose the two dependent parameters, *A*_*inter*_ and *A*_*intra*_, as the variables in the parameter space. Note that in the calculation, only the *s*-wave contact interaction on the *repulsive* side is taken into account, that is, both *A*_*inter*_ and *A*_*intra*_ are set to be positive. Our results suggest that there are three phase regions whose boundaries show nearly in linear pattern. Moreover, a triple point is found at *A*_*inter*_ = *A*_*intra*_ = 1.08 in the diagram, where three phases coexist. Further, our results show that the CS-II pattern takes place only if *A*_*intra*_ is large enough, and that for larger *A*_*inter*_, a larger critical value of *A*_*intra*_ is needed to trigger the FM transition. This implies that the coupling between spin 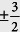 and 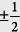 components suppresses the occurrence of FM phase.

**Figure 6:**
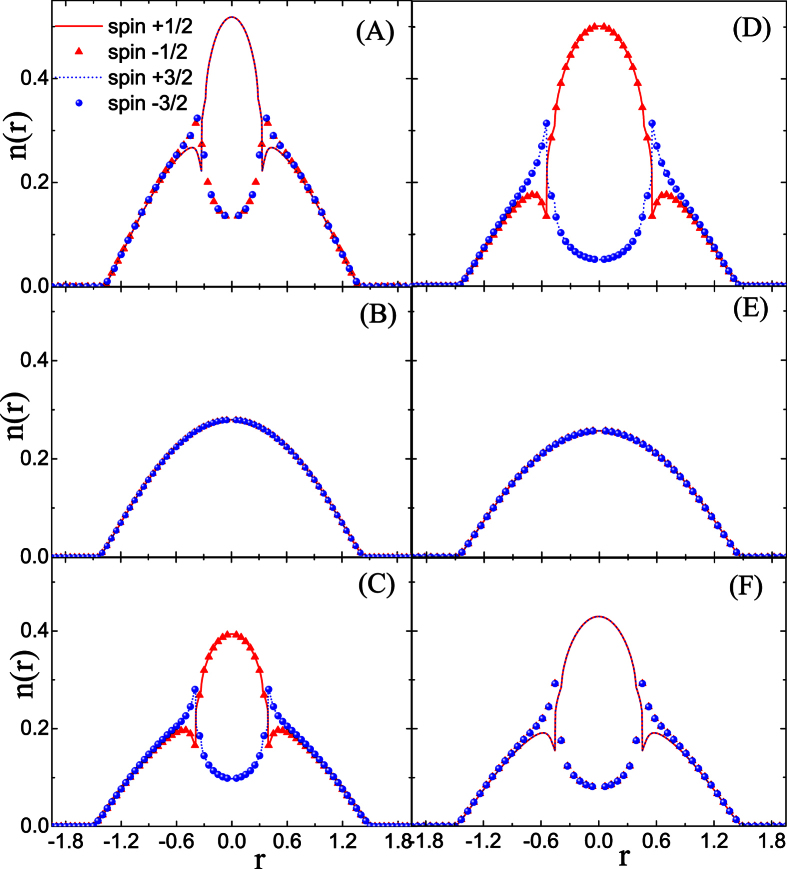
Density profiles of spin components in the LSFG under different conditions of *A*_*inter*_ and *A*_*intra*_, which corresponds to the points (A–F) marked in the phase diagram. Specifically, Points (**A–C**) correspond respectively to (*A*_*inter*_, *A*_*intra*_) = (0.3, 1.0), (0.6, 1.0), (1.05, 1.0), while points (**D–F**) correspond respectively to (*A*_*inter*_, *A*_*intra*_) = (0.9, 0.7), (0.9, 0.9), (0.9, 1.1).

The distribution of the phase regions can be easily understood according to the energetic analyze about Eq. (6). When the coupling strength *A*_*inter*_ (*A*_*intra*_) is increased, its corresponding contribution to the total ground energy is also enhanced, which prefers to trigger the CS-I(II) pattern because their advantage in the competition with the kinetic energy. To clarify the separation in the phase diagram, we choose six points (A)-(F) in different phase regions. Density profiles correspond to these points are plotted in Fig. 6, which shows the structure evolution from one pattern to another, when one coupling parameter varies while the other keeps unchanged.

### Coupling with *A*_*mix*_ ≠ 0

Compared to the above investigation with *A*_*mix*_ = 0, more information is expected when the spin mixing collision is taken into account. From the energetic point of view, the spin mixing opens another channel for the spin components to lower the total energy. Therefore, the effect of the spin mixing on the CS in the LSFG should be investigated. Before performing calculation, a quality, say 
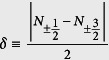
, is firstly defined to describe the amount of the atoms that change their spin quantum number from 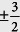 to 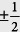. Accordingly, the ground state with spin mixing can be determined by comparing the total ground energy of the system with different values of *δ*.

**Figure 7:**
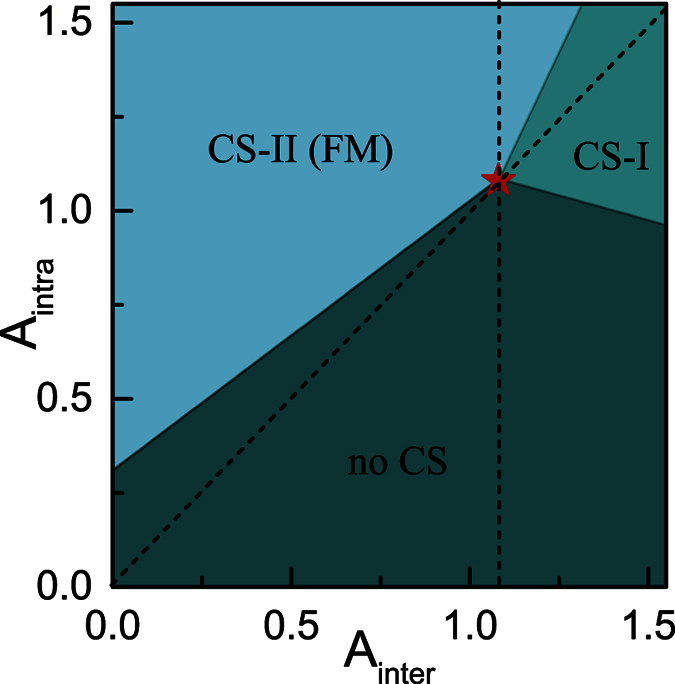
Phase diagram the LSFG with *A*_*mix*_ ≠ 0. The star point *S* represents the stationary triple-point which locates at (1.08, 1.08) in the diagram. The diagonal dash line is the dividing line corresponding to *A*_*inter*_ = *A*_*intra*_, while the vertical dash line passes through the triple-point *S*.

Note that in the calculation, two schemes have been employed to obtain the ground state densities. One is to assume that the separation of the CS-I pattern, while the other assume it of the CS-II pattern. The ground state is determined by comparing the total energy of the two patterns. Following this routine, the calculation is performed for the phase diagram, which shows clearly the effect of spin mixing collision on the phase separation. The phase diagram for *A*_*mix*_ ≠ 0 is presented in Fig. 7. Comparing with the results for the case of *A*_*mix*_ = 0 in Fig. 5, it is obvious that the boundary lines of the phase region have been rotated around the triple-point *S*(1.08, 1.08), which is stationary due to the relationship between *A*_*mix*_ and the other two coupling parameters: *A*_*mix*_ = 2(*A*_*intra*_ − *A*_*inter*_).

The hidden physics in the diagram can be understood as follow. Firstly, the CS-II pattern separation occurs only in the region above the diagonal dash line, which is in accordance with the case of *A*_*mix*_ = 0 shown in Fig. 5. This is because in this region, the energy contribution from intra-component collision dominates over that from inter-component collision. Secondly, for a given *A*_*inter*_ < 1.08, the critical value of *A*_*intra*_ has been declined because of the introduction the spin mixing collision, which helps the intra-component repulsion in its competition with the kinetic energy. This effect is even more significant especially for a smaller value of *A*_*inter*_, because it leads to a larger *A*_*mix*_. Therefore, the spin mixing collision is benefit to the occurrence of FM transition. Thirdly, for the given *A*_*inter*_ > 1.08, the spin mixing collision leads also to significant effect on the critical value of *A*_*intra*_ that triggers the CS-I pattern separation. That is, the CS-I pattern separation does not take place for all values of *A*_*inter*_ < 1.08. Moreover, with the increase of *A*_*inter*_, a smaller value of *A*_*intra*_ is required to triggers the CS-I pattern separation. This is because in the lower-right region of the parameter space, the fact *A*_*inter*_ > *A*_*intra*_ indicates that *A*_*mix*_ should be negative, which results in the decrease of the total energy. From this point of view, the spin mixing collision tends to suppress the CS-I pattern separation.

### Summary

In this work, the component separation in trapped LSFG is studied within the framework of density functional theory. The ground state density profile of each spin component is calculated. Our calculation suggests that when the spin mixing collision is absent, two patterns of CS take place, among which only the pattern-II separation corresponds to the itinerant FM state because of the formation of the local polarization in this case. Phase diagram shows that the coupling parameters *A*_*inter*_ and *A*_*intra*_ relate respectively to CS of pattern-I and pattern-II. On the other hand, when the spin mixing is taken into account, the phase distribution in the parameter space changes due to the newly opened collision channel. The phase diagram shows the interplay between the CS and spin exchange. That is, the spin exchange benefits the occurrence of the CS-II pattern separation, while it suppresses the CS-II pattern separation. Therefore, the spin mixing collision in LSFG plays a positive role in the detection of FM phase. It is hoped that our results can provide useful insight for the investigation of the FM transition in LSFG.

As an end for this section, we comment on the experimental feasibility of the observation of the CS in LSFG. Actually, the experimental setup for spin dynamics in LSFG^33^,^34^,^35^,^36^ can be shared to examine the results in this work. The system can be initially prepared with a balanced spin mixture with 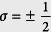, which is confined in a harmonic trap, and evaporated to a temperature 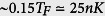. To access the off-diagonal element of the density matrix, short radio-frequency pulses should be applied, which rotates the spin with *ϑ* in spin space, and then results in a coupling of all possible spin components, whose magnetic quantum numbers ranges from *m* = −*F*, _···_, *F*. Finally, the Stern-Gerlach method can be used to determine the diagonal element^45^, which contains information of the off-diagonal element because they relate with each other through the rotation matrix.

## Methods

In this section, we give some details about the construction of the energy functional. As shown in Eq. (2), the interaction Hamiltonian has been decomposed into three parts, i.e., *H*_*inter*_, *H*_*intra*_ and *H*_*mix*_. Firstly, we calculate the expectation of *H*_*inter*_:


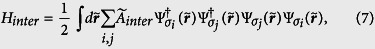


with 
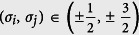
. Takes the channel between 
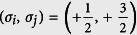
, one of the terms in the summation yields:


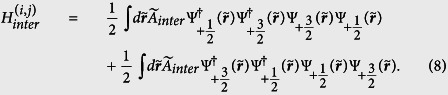


The product of the four field operators for the interacting Fermi atoms can be treated based on the Wick’s theorem:


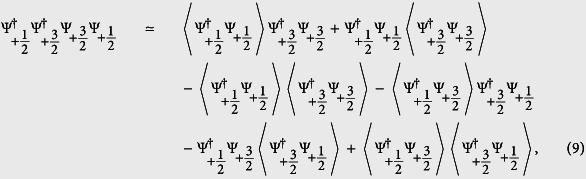


where 〈···〉 represents calculation of the expectation value of the inner. Further calculation the expectation of 
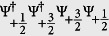
 yields:


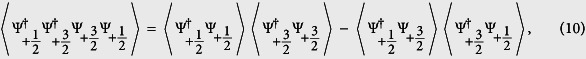


where the second term on the right-hand side describes the correlation between atoms belong to different spin components. In fact, in most of the studies on the two-components Fermi gas, the second term in Eq. (10) is usually ignored, and the qualitatively correct result can be achieved in prediction for FM transition^6^,^23^,^44^. Therefore, it is hoped that the extension of such a treatment to the study of LSFG can also give *qualitative* results, though the total energy has been overestimated by doing this. Introducing the local density operator 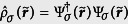, and assuming each spin component is highly occupied, the expectation of *H*_*inter*_ is obtained as:


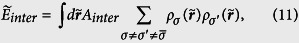


where the density operator has been replaced by the real density function. This result is in accordance with that from the first order perturbative approximation. Following a similar procedure, the expectation of *H*_*intra*_ can also be obtained as:


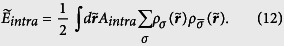


Further, we turn to the contribution from the spin mixing collision. Under the constraint of the conservation of the total angle momentum, spin mixing collision in LSFG permits different inert states in the incoming and outgoing channels. Assuming that only the *s*-wave (*l* = 0) scattering takes place, this part of contribution, *H*_*mix*_, can be rewritten as:


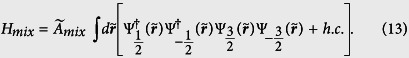


The spin mixing channel is unique to high spin fermions, which does not appear in the spin-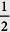 system. So it is expected that this term may cause nontrivial effect to itinerant ferromagnetism of the high spin fermions. However, this term can not be treated directly within the conventional density functional theory. We need adopt some approximation. In this work, in order to introduce the spin mixing contribution to the energy functional, we replace the field operators, as an *attempt*, with the square root of the density operators, i.e., 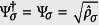. Further, replacing the density operators with the corresponding real density functions, the energy contribution with respect to *H*_*mix*_ can be respectively given as:


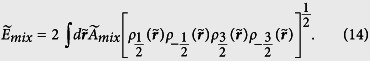


Combining the Eqs (11), (12), (14), we arrive at the total energy functional given in Eq. (3).

## Additional Information

**How to cite this article**: Sun, Z. and Gu, Q. Spontaneous separation of large-spin Fermi gas in the harmonic trap: a density functional study. *Sci. Rep.* **6**, 31776; doi: 10.1038/srep31776 (2016).
